# Applying a New Framework for Public Health Systems Recovery following Emergencies and Disasters: The Example of Haiti following a Major Earthquake and Cholera Outbreak

**DOI:** 10.4269/ajtmh.16-0862

**Published:** 2017-10-18

**Authors:** David L. Fitter, Daphnée Benoit Delson, Florence D. Guillaume, Angela Wood Schaad, Daphne B. Moffett, Jean-Luc Poncelet, David Lowrance, Richard Gelting

**Affiliations:** 1 Centers for Disease Control and Prevention, Port-au-Prince, Haiti;; 2 Ministry of Public Health and Population, Port-au-Prince, Haiti;; 3 Management Sciences for Health (MSH), Port-au-Prince, Haiti;; 4 Centers for Disease Control and Prevention, Atlanta, Georgia;; 5 Centers for Disease Control and Prevention, Almaty, Kazakhstan;; 6 Pan American Health Organization, Port-au-Prince, Haiti;; 7 Centers for Disease Control and Prevention, Dar es Salaam, Tanzania

## Abstract

Emergencies can often directly impact health systems of an affected region or country, especially in resource-constrained areas. Health system recovery following an emergency is a complex and dynamic process. Health system recovery efforts have often been structured around the World Health Organization’s health systems building blocks as demonstrated by the Post-Disaster Needs Assessment. Although this structure is valuable and well known, it can overlook the intricacies of public health systems. We retrospectively examine public health systems recovery, a subset of the larger health system, following the 2010 Haiti earthquake and cholera outbreak, through the lens of the 10 essential public health services. This framework illustrates the comprehensive nature of and helps categorize the activities necessary for a well-functioning public health system and can complement other assessments. Outlining the features of a public health system for recovery in structured manner can also help lay the foundation for sustainable long-term development leading to a more robust and resilient health system.

## INTRODUCTION

Emergencies often have a direct impact on the health systems and public health systems of an affected region or country, particularly in resource-constrained areas.^1–5^ The effects of an emergency on the performance and capacity of these systems depend upon a variety of interrelated factors, which include the predisaster status of the systems, the type of emergency, the effectiveness of the response, and the initiation of recovery activities.^6,7^ Health system recovery following an emergency is an intricate and dynamic process. The response is often still occurring as the steps toward recovery begin, although during this period recovery work is not recognized as the primary focus.^8^

**Figure 1.**
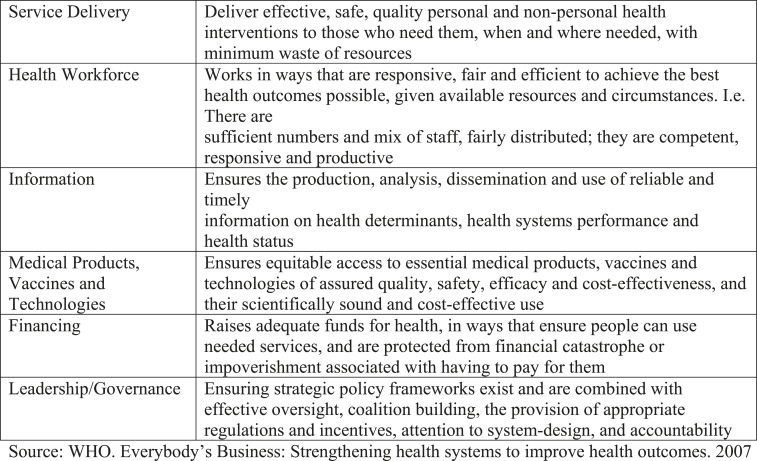
The World Health Organization (WHO) Health Systems Framework. Source: WHO.^9^

**Figure 2.**
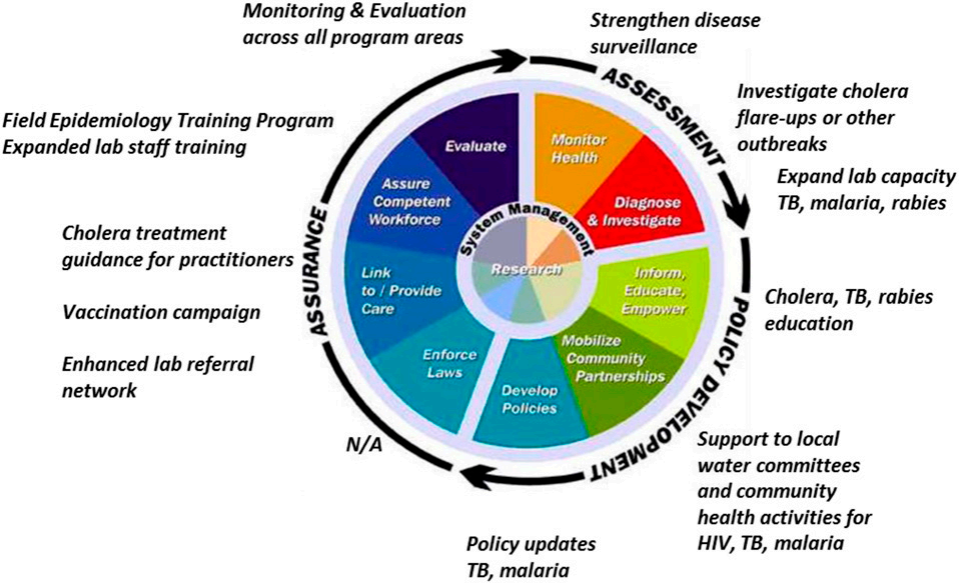
The 10 essential public health services and Centers for Disease Control and Prevention Health Systems Recovery Program in Haiti. Source: CDC.^11^ This figure appears in color at www.ajtmh.org.

The strategic objectives and operating procedures used to guide, develop, and initiate health system response and recovery plans are often framed by the World Health Organization (WHO) health system building blocks (Figure 1^9^), which are primarily used to inform health development priorities and plans. For example, the health subsector of WHO’s Post-Disaster Needs Assessment (PDNA) guidelines focuses on this framework.^10^ Although this structure has proven quite useful and there is extensive familiarity with it, it can overlook the intricacies of public health systems. In addition, the building blocks have often been used to characterize health systems rather than analyze them. To highlight public health system recovery, we retrospectively examined recovery work in Haiti through the framework of the 10 essential public health services (EPHSs) (Figure 2^11^). We used this alternate framework to illustrate the comprehensive nature of the public health systems recovery effort undertaken by the Haitian Ministry of Public Health and Population (MSPP) in conjunction with the U.S. Centers for Disease Control and Prevention (CDC), other U.S. government (USG) and United Nations agencies, as well as nongovernmental organizations, private foundations, and other bilateral organizations in response to the earthquake and cholera outbreak in Haiti in 2010. In addition, the EPHS can provide a complementary approach to the PDNA or other assessments, which can add value to current frameworks. We also discuss the applicability of the EPHS to a global context and how they relate to other frameworks that have applicability to recovery settings.

### Recovery and public health services.

Early recovery work aims to accompany response activities by capitalizing on existing emergency and humanitarian programs, supporting community initiatives, and establishing a path for longer term recovery.^12–15^ Early recovery also complements the response in addressing emergent postdisaster issues that existing health plans may no longer adequately address. Existing country programs may need to be reviewed in the context of new and evolving postdisaster priorities, to create new operational strategies.^12,16^ Transitional strategies for the reinvigoration of disrupted health services need to be implemented, while reexamining longer term operational plans.^8,16^ Nonetheless, the movement between relief, recovery, and development is fluid, especially in fragile states where emergencies may be frequent or recurring.^17^ This is evident in Haiti as well, where the recent Humanitarian Response Plan states that the transition from response to longer term programming “should be seen as a convergence process rather than sequential since the humanitarian and development needs occur simultaneously.”^18^

WHO has developed and expanded on a general framework for describing health systems composed of six building blocks including service delivery, health workforce, health information, medical commodities (including products, vaccines, and technology), financing, and leadership and governance (Figure 1).^19,20^ Although the WHO framework incorporates essential public health systems to some extent, further emphasis on public health services and programs is needed, especially in the context of recovery. That emphasis needs to account for many factors that influence the organization, priorities, quality, and performance of a public health system. These factors are often driven by a country’s history, the broader political climate, socioeconomic conditions, governance, and in some cases, existing internal capacity to respond and recover from disasters.^18^ Each country has unique obstacles in strengthening its public health system and must decide how best to prioritize and address them. In the case of Haiti, a history of instability, insecurity, and recurring emergencies has contributed to increased vulnerability and this context must be kept in mind. Involvement of national and local governments is therefore also essential to developing suitable recovery plans.^8,10,12^ As previously indicated, we used the EPHS framework in this paper to retrospectively examine issues related to public health system recovery in Haiti (Figure 2).

### Public health systems recovery in Haiti.

On January 12, 2010, Haiti was struck by a 7.0-magnitude earthquake, which was estimated to have killed over 200,000 people, left millions in need of urgent medical attention, and devastated an already weak national infrastructure.^10^ More than 1.5 million people were displaced when their homes were destroyed, raising concern for increased risk of epidemic-prone diseases.^21^ Although disease outbreaks do not spontaneously follow natural disasters, risk can be increased given such factors as displaced populations, poor water and sanitation, and decreased access to health services.^22,23^ However, this did not occur immediately in spite of the size of the movement of population, the environmental condition, or the high incidence level of most common communicable diseases.

A large-scale response to the earthquake was mounted that involved multiple organizations and included the activation of the health cluster coordination group, a coordinating group for both local and international organizations that is typically led by the host country ministry of health and Pan American Health Organization (PAHO)/WHO. Less than a year after the earthquake, Haiti was hit by another disaster. It had its first case of cholera suspected by Cuban brigades and PAHO on October 18 and confirmed by the Haitian National Public Health Laboratory on October 21, 2010.^24^ The cholera outbreak placed further stress on an already fragile health system in the early stages of recovery following the earthquake. The lack of water and sanitation infrastructure and services compounded by the weakness of the system and the economy were the main contributors to the widespread epidemic.^25,26^

Several recovery plans were developed in the wake of these two emergencies. Following the earthquake, the government of Haiti, with support from the international community, developed an Action Plan for National Recovery and Development, which was based on the PDNA and other considerations.^27,28^ The cholera outbreak in October 2010 saw a similar collaboration between the Haitian government and partners with the development of a specific national plan for the elimination of cholera.^29^ In keeping with the PDNA process, these plans included elements of risk reduction to increase resilience. In the public health arena, MSPP, in close collaboration with key national and international partners within the health cluster, developed recovery plans focused on national public health issues of importance that were also linked to previous government health strategies. Immediate public health priorities for cholera concentrated on improving surveillance, water and sanitation, alert and response, and laboratory capacity to assist with detection and response of cholera and other public health threats.^13,25,30^ Additional public health priorities were determined by examining public health programs in need of investment in the medium to long-term time frame.^31,32^ Developing workforce and emergency response capacity within public health were some of the areas included in these longer term efforts. Although disease burden was a factor in determining areas of focus, it was not the only consideration, as MSPP priorities, including expanding access to public health services for all Haitians, also influenced the process.^33,34^ Furthermore, MSPP sought to increase collaboration across programs recognizing the value of integrating public health services and leveraging the limited resources available in the country.^35^ A body within MSPP was entrusted with the coordination of the cholera response. Finally, the Millennium Development Goals were an additional reference point in the development of recovery plans, underscoring the fundamental interconnection between response and longer term programming, even in fragile situations where recurrent emergencies occur.

The PDNA and other assessments, allowed the Haitian government, along with key stakeholders to develop a public health recovery strategy addressing surveillance, laboratory strengthening, and water, sanitation and hygiene (WASH), in addition to longstanding public health development challenges the country had faced before the earthquake and cholera outbreak, such as tuberculosis (TB), human immunodeficiency virus, and maternal and child health.^36^ Stakeholders, including MSPP, the PAHO, WHO, USG, and other partners, participated in defining high-level public health goals to improve the public health system in Haiti through postearthquake and cholera recovery efforts.^37,38^ This work was initially done through the health cluster, which at the beginning of the response was led by PAHO/WHO.^39^ This cluster approach resulted in sector-specific gap analyses, which were used to guide MSPP and partners through the process of strategic planning that aligned goals and defined qualitative and quantitative indicators and implementation targets. This approach assisted in defining priorities, mobilizing stakeholders, targeting funding, informing program development, and monitoring implementation.^36^ There were challenges with the cluster system, including initial coordination and information management, but there were also eventual successes as seen with surveillance and WASH.^40^ As recovery progressed over the years, coordination transitioned from the health cluster to MSPP and longer term goals were jointly developed to further focus resources and align priorities as is seen in the Haiti Health Plan, 2012–2022.^34^ These goals were broadly disseminated to encourage, with limited success, coordination of resources by partners investing in health in Haiti. In addition, programs examined where resources could be shared and aligned to maximize impact, in line with recent studies examining the side effects of disease-specific programs that may have the unintended consequence of decreasing overall health system capacity with a focus on vertical systems.^41^

### A framework for public health systems recovery.

The public health recovery efforts undertaken by MSPP and other partners in response to both disasters in Haiti were intended to achieve the broader goal of strengthening the health system and core public health services. These services can be seen as those fundamental actions performed to address the determinants of health with the aim to improve, promote, protect, and restore the health of the population through the organized efforts of society.^42,43^ For the purposes of this paper, we examine public health recovery efforts in Haiti through the lens of CDC’s EPHS framework. This was done retrospectively to examine the utility of this framework in complementing assessments such as the PDNA. The EPHS were not used to structure activities during recovery, although there was a conscious effort by MSPP and partners to ensure that a broadly comprehensive approach was taken. The EPHS “describe the public health activities that all communities should undertake,” and provide both a conceptual basis and reference for defining and organizing key activities necessary for a well-functioning public health system.^11^

Figure 2 illustrates the three core functions (assessment, policy development, and assurance), and the 10 essential services of public health as outlined in the EPHS.^44,45^ Research is one of the essential services, as well as a cross-cutting activity that, along with systems management, spans across the core functions and other essential services. Recovery efforts in Haiti covered virtually the entire spectrum of these public health activities, as outlined in Table 1. This is by no means an exhaustive list; however, its purpose is to highlight examples of key aspects of the public health recovery effort as they align with the EPHS. Although these efforts have been summarized elsewhere,^30,31^ the purpose of this paper is to place them within a comprehensive public health framework illustrating the integrated nature of the recovery program in Haiti. Figure 2 also illustrates the broad range of recovery activities that CDC and other partners supported in Haiti and demonstrates how they align closely with the core functions and essential services, helping to maximize the impact of recovery activities as program efforts returned to development. For example, within the assessment phase, strengthening disease surveillance and laboratory capacity were key elements of recovery and development; this included a variety of diseases beyond cholera. In the policy development phase, health education was crucial for prevention, both at the community level for cholera, and in health-care facilities for TB. Within the assurance phase, multiple efforts aimed at strengthening public health workforce capacity were implemented, including the establishment of the Field Epidemiology Training Program to train Haitian epidemiologists.^36^ Developing workforce capacity also took place through helping establish other new programs, such as the Rural Water and Sanitation Technicians for the Communes of the National Directorate for Potable Water and Sanitation (DINEPA)^46^ and providing cholera treatment guidance for practitioners initially unfamiliar with the disease. Leveraging existing system financial and administrative management capacity was also included, as exemplified by the financial oversight provided by MSPP through the program management unit originally set up under the President’s Emergency Plan for AIDS Relief program and expanded for response and recovery work. Research was an integral aspect of multiple programs such as vaccine effectiveness and coverage studies included in oral cholera vaccination efforts.

**Table 1:** Recovery Programs examples in Haiti and the 10 EPHS

Core function	EPHS	Recovery activities in Haiti
Assessment	Monitor health	Launched national syndromic surveillance system in health facilities and IDP camps
		Established national laboratory-enhanced sentinel surveillance system linking laboratory testing for priority diseases (e.g., measles, rubella, diphtheria) to epidemiologic data
		Strengthened/established disease/condition-specific surveillance activities, including for cholera, malaria, maternal health, rabies, and TB
	Diagnose and investigate	Identification/investigation of cholera flare-ups and other outbreaks
		Development of response plans for cholera flare-ups and Ebola preparedness
		Improved laboratory capacity to diagnose TB, malaria, rabies
Policy development	Inform, educate, empower	Community health education/promotion, including by mass media, on cholera prevention, including WASH
		Health-care worker training on TB, including infection, prevention, and control
	Mobilize community partnerships	Support to local water committees managing rural piped drinking water systems through DINEPA
		Established regional coalition for water and sanitation to eliminate cholera in Hispaniola, with membership from over 20 international organizations
		Funding of community health activities for HIV, TB, malaria
	Develop policies	Revised national policies on malaria diagnosis and case management
		Assisted in development of national policy/procedures on TB testing
Assurance	Enforce laws	No specific activities are included here, but technical assistance to develop policies provided
	Link to/provide care	Provided guidance and training on cholera treatment guidance for practitioners initially unfamiliar with the disease
		Rapid restoration and expansion of HIV/AIDS treatment services following earthquake
		Implemented and evaluated national measles-rubella vaccination campaign
		Greatly expanded mass drug administration against lymphatic filariasis
		Enhanced laboratory specimen referral network, which has included HIV, TB, cholera, and other disease-surveillance specimens
	Assure competent workforce	Established Field Epidemiology Training Program, resulting in over 200 graduates that have worked on cholera, dengue, and chikungunya outbreaks in Haiti
		Established Rural Water and Sanitation Technicians for the Communes program within the National Potable Water and Sanitation Directorate, placing over 250 technicians in all communes outside metropolitan Port-au-Prince
		Trained laboratory staff on testing for TB, rabies, malaria
		Established GIS capacity within MSPP
	Evaluate	Evaluation of immunization campaigns, including measles-rubella and oral cholera vaccines
		Evaluated KAP related to cholera in Artibonite Department
Cross-cutting across all core functions	Research	Compared RDTs and microscopy for malaria diagnostic testing
		Conducted vaccine effectiveness and coverage studies for OCV
	Systems management	Strengthened management capacity in MSPP and DINEPA through technical assistance and funding support
		Reinforced existing financial management capacity originally established within MSPP by PEPFAR

AIDS = acquired immune deficiency syndrome; DINEPA = Directorate for Potable Water and Sanitation; GIS = geographic information system; HIV = human immunodeficiency virus; IDP = internally displaced persons; KAP = knowledge, attitudes and practices; MSPP = Haitian Ministry of Public Health and Population; OVC = orphans and vulnerable children; PEPFAR = President’s Emergency Plan for AIDS Relief; RDT = rapid diagnostic test; TB = tuberculosis; WASH = water, sanitation and hygiene.

Resources were directed to MSPP and other Haitian government agencies such as DINEPA, as building their capacity was critical to long-term recovery and development efforts. Recovery efforts in Haiti were also designed to be multipurpose and mutually reinforcing. For example, strengthening laboratory-enhanced disease surveillance before and after the earthquake facilitated rapid detection and identification of the cholera outbreak.^25^ Similarly, training programs for health-care workers were designed to take advantage of cross-training opportunities to broaden expertise among staff, so they could support several programs at their sites, as opposed to only working on a single-funded program.^36^

The above discussion illustrates the integrated, inclusive, and “diagonal” approach to postdisaster public health system strengthening taken in Haiti.^47,48^ This method was designed to broadly strengthen public health programs in Haiti using a horizontal approach, while also allowing for integration of vertical, disease-specific programs that are commonly seen in public health. As such, it provides a potential example for structuring public health systems strengthening programs whether in the postdisaster setting or for routine development.

Recovery activities in Haiti predated the development of the Global Health Security Agenda (GHSA) and the Joint External Evaluation (JEE) process associated with GHSA. In addition, the JEE is designed more as an assessment rather than a tool for recovery. Nonetheless, the areas identified and activities undertaken during recovery in Haiti reflect public health priorities of GHSA, namely, strengthening surveillance, laboratory, and workforce capacity and improving emergency response.^49–51^ These same priorities reflect key elements related to public health that are included within the 2005 International Health Regulations (IHRs) core capacities, including surveillance, laboratory, preparedness, and response.^50,52^ This underscores the broad nature of the EPHS, which is to say that they can be used throughout response, recovery, and development to structure public health activities.

## DISCUSSION

The Haiti earthquake and cholera emergencies depended on a large coalition of national and international partners to assist in response and recovery efforts. This extensive action followed many of the suggested modalities for recovery work in a postemergency or disaster setting, as exemplified by the use of the PDNA to provide information for policy and strategy setting.^8^ In addition, plans from MSPP, such as the Action Plan for National Recovery and Development of Haiti and the Haiti Health Plan, 2012–2022,were established early that served as a basis for the strategic planning for recovery efforts.^27,34^ Many of the priorities helped reinforce the EPHSs of the health system. Six years after the earthquake, progress is evident in many areas as demonstrated by examples listed in Figure 2 and Table 1 and also documented elsewhere.^30,31,36^ However, Haiti faces yet another challenge in the aftermath of Hurricane Matthew.

Though the field of health systems recovery remains relatively nascent, several frameworks have been developed.^8,12,14,53^ However, many of these examine the broader health system in the context of the WHO building blocks, with considerable emphasis on service delivery systems. We have presented the EPHS as a complementary model focused on public health systems. We applied the EPHS to retrospectively examine the structure of public health system recovery activities in Haiti. This analysis indicated that public health system recovery in Haiti was comprehensive and broadly based and a broad spectrum of accomplishments was noted (see Figure 2 and Table 1). As indicated in the aforementioned documents on various frameworks for recovery, increased detail for the various recovery sectors is needed to tailor strong recovery plans toward an eventual reconvergence with long-term development programs with a prioritized strategic and operational approach that is well aligned with national and international health sector priorities. Developing such a plan-assisted MSPP, PAHO, CDC, and other partners to develop comprehensive and integrated activities and indicators, helping to lead to multiple successful outcomes.^30^ PAHO’s cooperation with Haiti was completely reviewed including major change in staff and funding for example in essential medicine, vaccination, or attention to pregnant women.

Within the context of public health system recovery work in Haiti, several lessons learned reinforce what has been documented elsewhere, but their importance merits being highlighted. Priority setting at a more refined level is key to ensuring the most relevant and impactful activities are implemented.^8,33^ This activity should take into account new, exigent priorities driven by the emergency in context with preexisting country objectives. The feasibility of implementing prioritized activities, including accounting for the capacity of implementing partners such as ministries of health, must also be examined when setting priorities. With reference to the EPHS, the significance of policy development, workforce, communication, and research was critical. The WHO European office, with their Essential Public Health Operations (EPHOs), has designated these as enabler functions, which are those functions that allow the other functions to operate.^54^

For broader application in the global context and potential use in other settings, some modifications to the EPHS would be needed and other frameworks can inform those changes. For example, the EPHS do not encompass governance and financing, both of which are crucial features for any system, as demonstrated by their importance in the WHO building blocks and the EPHOs. Governance is embedded within the EPHS through the cross-cutting element of system management, but may need to be elaborated and made more specific. All of these factors together aim to create a more resilient health system. In addition, any framework used to structure or analyze recovery activities, should ensure alignment with and integration of principles from IHR core capacities, as these have been become global guidelines for public health functions. As discussed earlier, the EPHS, as one potential framework, do incorporate many key elements from the IHR. Nonetheless, further alignment with the IHR would strengthen the framework in addition to making it more familiar to a global audience.

Stakeholder and implementing partner activities and timelines for implementation need to be regularly addressed. This is in line with the Inter-Agency Standing Committee (the primary mechanism for interagency coordination of humanitarian assistance and designation of clusters) on humanitarian assistance recommendations to use the 4Ws (Who, What, When, Where) to map activities from all partners.^55^ Although it was initially tailored to the response, similar recommendations can be made for recovery.^12^ An appropriate timeline will enable smoother handover to the government or another implementing partner and prevent avoidable gaps in activities and the provision of services.

A strong public health system recovery contributes to broader recovery and health development objectives as a whole. This complements the integrated process for disaster risk reduction. The response and recovery process are being seen as part of the disaster risk reduction continuum, which also includes preparedness, response, mitigation, and sustainable development.^56^ Capitalizing on the response and recovery process to enhance established development programs is more easily accomplished with strong, detailed frameworks from which countries and partners can work. The EPHS provide one potential model for such a framework, although, as discussed earlier, some modifications may be necessary for broad global applicability.

## CONCLUSION

Public health systems recovery from emergencies and disasters is complex. Nonetheless, when undertaken in a structured and transparent manner, it can help to lay a foundation for transitioning to sustainable long-term development and make public health systems more robust and resilient to future disasters.^57^ Recovery should build on and augment response programs while also complementing previous health system plans that were in place before an emergency or disaster. Ideally, planning for recovery begins during the response phase. Utilizing a framework such as the 10 EPHSs can help to structure public health recovery activities, and ensure that they are comprehensive and integrated, especially when implemented in conjunction with multiple stakeholders, including host governments. As Haiti begins its recovery from Hurricane Matthew, this type of framework may prove useful. Other frameworks such as the JEE process within the GHSA, which were not yet available during Haiti’s recovery from the 2010 emergencies could also be used to help ensure that recovery is undertaken in a structured and comprehensive manner. The EPHS were used in this analysis because they provided a straightforward, existing structure and method to retrospectively examine public health recovery in Haiti.

## References

[1] Spiegel PB Le P Ververs MT Salama P, 2007 Occurrence and overlap of natural disasters, complex emergencies and epidemics during the past decade (1995–2004). Confl Health 1: 2. 17411460 10.1186/1752-1505-1-2 PMC1847810

[2] Ivers LC, 2011 Strengthening the health system while investing in Haiti. Am J Public Health 101: 970–971. 21493927 10.2105/AJPH.2010.300108 PMC3093291

[3] Ben Taleb Z Bahelah R Fouad FM Coutts A Wilcox M Maziak W, 2015 Syria: health in a country undergoing tragic transition. Int J Public Health 60 (Suppl 1): S63–S72. 25023995 10.1007/s00038-014-0586-2

[4] Loretti A Tegegn Y, 1996 Disasters in Africa: old and new hazards and growing vulnerability. World Health Stat Q 49: 179–184. 9170231

[5] Kieny MP Dovlo D, 2015 Beyond Ebola: a new agenda for resilient health systems. Lancet 385: 91–92. 25706456 10.1016/S0140-6736(14)62479-X PMC5513100

[6] Ivers LC Ryan ET, 2006 Infectious diseases of severe weather-related and flood-related natural disasters. Curr Opin Infect Dis 19: 408–414. 16940862 10.1097/01.qco.0000244044.85393.9e

[7] de Ville de Goyet C Marti RZ Osorio C, 2006 Natural disaster mitigation and relief. Jamison DT, Breman JG, Measham AR, Alleyne G, Claeson M, Evans DB, Jha P, Mills A, Musgrove P, eds. *Disease Control Priorities in Developing Countries*. Washington, DC: The International Bank for Reconstruction and Development/The World Bank Group.

[8] GFDRR, 2015 Guide to Developing Disaster Recovery Frameworks. Available at: https://www.gfdrr.org/recovery-framework-0. Accessed April 5, 2016.

[9] World Health Organization, 2007 Everybody’s Business: Strengthening Health Systems to Improve Health Outcomes: WHO’s Framework for Action. Available at: http://www.who.int/healthsystems/strategy/everybodys_business.pdf. Accessed April 5, 2016.

[10] United Nations Development Programme, 2015 Post-Disaster Needs Assessments. Available at: http://www.undp.org/content/undp/en/home/librarypage/crisis-prevention-and-recovery/pdna.html. Accessed April 4, 2016.

[11] Centers for Disease Control and Prevention (CDC), 2014 The Public Health System and the 10 Essential Public Health Services. Available at: http://www.cdc.gov/nphpsp/essentialservices.html. Accessed March 23, 2015.

[12] Cluster Working Group on Early Recovery, 2008. *Guidance Note on Early Recovery*. Available at: http://www.undp.org/content/undp/en/home/librarypage/crisis-prevention-and-recovery/guidance-note-on-early-recovery-cwger-april-2008.html. Accessed April 4, 2016.

[13] World Bank, 2008 Good Practice Note - Health. Available at: http://siteresources.worldbank.org/CHINAEXTN/Resources/318949-1217387111415/Health_en.pdf. Accessed April 2, 2015.

[14] Federal Emergency Management Agency, 2011. *National Disaster Recovery Framework: Strengthening Disaster Recovery for the Nation*. Available at: https://www.fema.gov/pdf/recoveryframework/ndrf.pdf. Accessed October 29, 2016.

[15] World Health Organization, 2017 Health Cluster Chapter on Early Recovery and Resilience. Geneva, Switzerland: WHO.

[16] International Federation of the Red Cross, 2012 *IFRC Recovery Programming Guidance*. Available at: http://www.ifrc.org/PageFiles/41104/IFRC%20Recovery%20programming%20guidance%202012%20-%201232900.pdf. Accessed October 29, 2016.

[17] Irina Mosel SL, 2014 Remaking the Case for Linking Relief, Rehabilitation and Development. Available at: https://www.odi.org/publications/8319-relief-rehabilitation-development-resilience. Accessed April 26, 2016.

[18] de Meritens JR Lawry-White J Davies A Kefford S Handley S, 2016 *Guidance Note on Inter-Cluster Early Recovery*. Available at: http://earlyrecovery.global/sites/default/files/guidance_note_-010816_0.pdf. Accessed April 30, 2017.

[19] World Health Organization, 2000 World Health Report 2000. Health Systems Performance Assessment. Available at: http://www.who.int/whr/2000/en/. Accessed October 21, 2015.

[20] Pavignani E, 2009 Analysing Disrupted Health Systems: A Modular Manual. Geneva, Switzerland: World Health Organization.

[21] Polonsky J, 2013 Public health surveillance after the 2010 Haiti earthquake: the experience of medecins sans frontieres. *PLoS Curr 5:* pii: ecurrents.dis.6aec18e84816c055b8c2a06456811c7a.

[22] Floret N Viel JF Mauny F Hoen B Piarroux R, 2006 Negligible risk for epidemics after geophysical disasters. Emerg Infect Dis 12: 543–548. 16704799 10.3201/eid1204.051569 PMC3294713

[23] Watson JT Gayer M Connolly MA, 2007 Epidemics after natural disasters. Emerg Infect Dis 13: 1–5. 17370508 10.3201/eid1301.060779 PMC2725828

[24] Centers for Disease Control and Prevention, 2010 Cholera outbreak—Haiti, October 2010. MMWR Morb Mortal Wkly Rep 59: 1411. 21048563

[25] Barzilay EJ, 2013 Cholera surveillance during the Haiti epidemic—the first 2 years. N Engl J Med 368: 599–609. 23301694 10.1056/NEJMoa1204927

[26] Gelting R Bliss K Patrick M Lockhart G Handzel T, 2013 Water, sanitation and hygiene in Haiti: past, present, and future. Am J Trop Med Hyg 89: 665–670. 24106193 10.4269/ajtmh.13-0217 PMC3795096

[27] Government of the Republic of Haiti, 2010 Action Plan for National Recovery and Development of Haiti. Available at: http://www.haitireconstructionfund.org/system/files/Haiti%20Action%20Plan.pdf. Accessed April 3, 2015.

[28] Government of Haiti, 2010 Haiti Earthquake PDNA: Assessment of Damage, Losses, General and Sectoral Needs. Available at: http://www.gafspfund.org/sites/gafspfund.org/files/Documents/Haiti_PDNA.pdf. Accessed April 3, 2015.

[29] Republic of Haiti, 2013 National Plan for the Elimination of Cholera in Haiti 2013–2022. Available at: http://www.paho.org/hq/index.php?option=com_docman&task=doc_view&gid=20326&Itemid=270&lang=en. Accessed April 7, 2016.

[30] Domercant JW Guillaume FD Marston BJ Lowrance DW, 2015 Update on progress in selected public health programs after the 2010 earthquake and cholera epidemic: Haiti, 2014. MMWR Morb Mortal Wkly Rep 64: 137–140. 25695317 PMC4584701

[31] Vertefeuille JF Dowell SF Domercant JW Tappero JW, 2013 Cautious optimism on public health in post-earthquake Haiti. Lancet 381: 517–519. 23332167 10.1016/S0140-6736(13)60051-3

[32] Dowell SF Tappero JW Frieden TR, 2011 Public health in Haiti—challenges and progress. N Engl J Med 364: 300–301. 21219131 10.1056/NEJMp1100118

[33] McGregor S Henderson KJ Kaldor JM, 2014 How are health research priorities set in low and middle income countries? A systematic review of published reports. PLoS One 9: e108787. 25275315 10.1371/journal.pone.0108787 PMC4183511

[34] Haiti Ministry of Public Health and Population, 2012 *Plan Directeur de Santé, 2012–2022*. Available at: https://mspp.gouv.ht/site/downloads/Plan%20Directeur%20de%20Sante%202012%202022%20version%20web.pdf. Accessed April 7, 2016.

[35] Schuchat A De Cock KM, 2012 The value of science in integration of services. J Infect Dis 205 (Suppl 1): S1–S3. 22315376 10.1093/infdis/jir801

[36] CDC, 2013 Progress Toward Rebuilding Haiti’s Health System. Available at: http://www.cdc.gov/globalhealth/healthprotection/errb/pdf/progresstowardrebuildinghaitishealthsystem.pdf. Accessed April 7, 2016.

[37] Centers for Disease Control and Prevention, 2010 Rapid establishment of an internally displaced persons disease surveillance system after an earthquake—Haiti, 2010. MMWR Morb Mortal Wkly Rep 59: 939–945. 20689498

[38] World Bank, 2010 Haiti Earthquake PDNA: Assessment of Damage, Losses, General and Sectoral Needs. Available at: http://documents.worldbank.org/curated/en/2010/03/16394500/haiti-earthquake-pdna-post-disaster-needs-assessment-assessment-damage-losses-general-sectoral-needs. Accessed April 26, 2016.

[39] Dhillon P Annunziata G, 2012 The Haitian health cluster experience: comparative evaluation of the professional communication response to the 2010 earthquake and the subsequent cholera outbreak. PLoS Curr 4: e5014b1b407653. 10.1371/5014b1b407653 PMC3470478 23074693

[40] Claude de Ville de Goyet JPS, Francois Grunewald, 2011. *Health Response to the Earthquake in Haiti: January 2010. Lessons to be Learned for the Next Massive Sudden-Onset Disaster*. Available at: http://new.paho.org/disasters/dmdocuments/HealthResponseHaitiEarthq.pdf. Accessed April 30, 2017.

[41] World Health Organization, 2008 Maximizing positive synergies between health systems and Global Health Initiatives. Available at: http://www.who.int/healthsystems/GHIsynergies/en/. Accessed April 1, 2015.

[42] Laaser U Brand H, 2014 Global health in the 21st century. Glob Health Action 7: 23694. 24560267 10.3402/gha.v7.23694 PMC3926989

[43] Pan American Health Organization, 2008 *The Essential Public Health Functions as a strategy for improving overall health systems performance: Trends and challenges since the Public Health in the Americas Initiative, 2000–2007*. Available at: http://www1.paho.org/hq/dmdocuments/2010/EPHF_Strategy_to_Strengthen_Performance.pdf. Accessed April 30, 2017.

[44] Corso LC Wiesner PJ Halverson PK Brown CK, 2000 Using the essential services as a foundation for performance measurement and assessment of local public health systems. J Public Health Manag Pract 6: 1–18. 10.1097/00124784-200006050-00003 11067656

[45] Institute of Medicine Committee for the Study of the Future of Public Health, 1988 *The Future of Public Health*. Washington, DC: National Academies Press (US), Copyright(c) 1988 by the National Academy of Sciences.

[46] Hubbard B, Lockhart G, Gelting RJ, Bertrand F, 2014 Development of Haiti’s rural water, sanitation and hygiene workforce. J Water Sanit Hyg Dev 4: 159–163. 10.2166/washdev.2013.089 PMC6889958 31798827

[47] Kim JY Farmer P Porter ME, 2013 Redefining global health-care delivery. Lancet 382: 1060–1069. 23697823 10.1016/S0140-6736(13)61047-8

[48] Sepúlveda J, 2006 Disease Control Priorities in Developing Countries, 2nd edition Washington, DC: World Bank.

[49] Ijaz K Kasowski E Arthur RR Angulo FJ Dowell SF, 2012 International Health Regulations—what gets measured gets done. Emerg Infect Dis 18: 1054–1057. 22709593 10.3201/eid1807.120487 PMC3376826

[50] World Health Organization, 2008 International Health Regulations 2005, 2nd edition Available at: http://apps.who.int/iris/bitstream/10665/43883/1/9789241580410_eng.pdf. Accessed April 5, 2016.

[51] World Health Organization, 2013 Checklist and Indicators for Monitoring Progress in the Development of IHR Core Capacities in States Parties. Available at: http://www.who.int/ihr/checklist/en/. Accessed April 5, 2016.

[52] Katz R Sorrell EM Kornblet SA Fischer JE, 2014 Global health security agenda and the international health regulations: moving forward. Biosecur Bioterror 12: 231–238. 25254911 10.1089/bsp.2014.0038

[53] Committee on Post-Disaster Recovery of a Community’s Public Health Medical, and Social Awareness, Board on Health Sciences Policy, Institute of Medicine, 2015. *Healthy, Resilient, and Sustainable Communities After Disasters: Strategies, Opportunities, and Planning for Recovery*. Washington, DC: National Academy of Sciences. 26401544

[54] Europe WHO, 2012 The 10 Essential Public Health Operations. Available at: http://www.euro.who.int/en/health-topics/Health-systems/public-health-services/policy/the-10-essential-public-health-operations. Accessed April 8, 2016.

[55] IASC, 2003 Exit Strategy for Humanitarian Actors in the Context of Complex Emergencies. Available at: https://interagencystandingcommittee.org/system/files/legacy_files/exitstrategy.doc.pdf. Accessed April 27, 2016.

[56] Global Facility for Disaster Reduction and Recovery, 2015 *Resilient Recovery: An Imperative for Sustainable Development*. Available at: https://www.gfdrr.org/sites/default/files/publication/wrc2_proceedings_7_28_2015.pdf. Accessed October 30, 2016.

[57] UNISDR, 2010 Guidance Note on Recovery: Health. Available at: https://www.unisdr.org/we/inform/publications/18782. Accessed April 26, 2016.

